# Engineered *Thermoanaerobacterium aotearoense* with *nfnAB* knockout for improved hydrogen production from lignocellulose hydrolysates

**DOI:** 10.1186/s13068-019-1559-8

**Published:** 2019-09-10

**Authors:** Yang Li, Jialei Hu, Chunyun Qu, Lili Chen, Xiaolong Guo, Hongxin Fu, Jufang Wang

**Affiliations:** 1 School of Biology and Biological Engineering, South China University of Technology, Guangzhou, 510006 China; 2 State Key Laboratory of Pulp and Paper Engineering, South China University of Technology, Guangzhou, 510640 China

**Keywords:** Metabolic engineering, *Thermoanaerobacterium aotearoense* SCUT27, NfnAB, Biohydrogen, Lignocellulose hydrolysate

## Abstract

**Background:**

As a renewable and clean energy carrier, the production of biohydrogen from low-value feedstock such as lignocellulose has increasingly garnered interest. The NADH-dependent reduced ferredoxin:NADP^+^ oxidoreductase (NfnAB) complex catalyzes electron transfer between reduced ferredoxin and NAD(P)^+^, which is critical for production of NAD(P)H-dependent products such as hydrogen and ethanol. In this study, the effects on end-product formation of deletion of *nfnAB* from *Thermoanaerobacterium aotearoense* SCUT27 were investigated.

**Results:**

Compared with the parental strain, the NADH/NAD^+^ ratio in the *∆nfnAB* mutant was increased. The concentration of hydrogen and ethanol produced increased by (41.1 ± 2.37)% (*p* < 0.01) and (13.24 ± 1.12)% (*p* < 0.01), respectively, while the lactic acid concentration decreased by (11.88 ± 0.96)% (*p* < 0.01) when the *∆nfnAB* mutant used glucose as sole carbon source. No obvious inhibition effect was observed for either SCUT27 or SCUT27/*∆nfnAB* when six types of lignocellulose hydrolysate pretreated with dilute acid were used for hydrogen production. Notably, the SCUT27/*∆nfnAB* mutant produced 190.63–209.31 mmol/L hydrogen, with a yield of 1.66–1.77 mol/mol and productivity of 12.71–13.95 mmol/L h from nonsterilized rice straw and corn cob hydrolysates pretreated with dilute acid.

**Conclusions:**

The *T. aotearoense* SCUT27/*∆nfnAB* mutant showed higher hydrogen yield and productivity compared with those of the parental strain. Hence, we demonstrate that deletion of *nfnAB* from *T. aotearoense* SCUT27 is an effective approach to improve hydrogen production by redirecting the electron flux, and SCUT27/*∆nfnAB* is a promising candidate strain for efficient biohydrogen production from lignocellulosic hydrolysates.

## Background

Fossil fuels such as natural gas, coal and petroleum have dominated the energy supply for long time. In 2017, renewable energy sources accounted for < 3% of the worldwide primary energy supply, while fossil fuels accounted for > 80% [1]. The use of carbon-based nonrenewable fossil fuels causes serious environmental problems. To address these problems, many efforts have been made to explore and produce renewable and environmental-friendly energy [2, 3], especially biofuels (e.g., hydrogen, bioethanol and methane).

Biofuels can be produced from biomass via biological or thermochemical processes and they generally exist in liquid (biodiesel and bioethanol) or gaseous form (biohydrogen and biomethane) [4]. The production of biofuels using corn, wheat and sugarcane results in a substantial increase of food prices, and nonfood lignocellulosic resource could be an alternative to solve this problem [5, 6]. As clean energy carriers, hydrogen has the potential to replace traditional fuels because of its high energy capacity and environmental friendliness [7, 8]. In 2018, the main sources for hydrogen were natural gas (approximate 48%), oil (30%) and coal (18%), while only 1.0% of hydrogen was derived from the conversion of biomass by microorganisms [9–11]. There are four hydrogen-producing methods using organisms, including the microbial electrolysis cell [12], biophotolysis [13], photofermentation [14], and dark fermentation [15–17]. Dark fermentation has been a research focus because of its wide substrate range, simple operation and easy industrialization, but it still suffers from major technical problems such as high substrate cost and low hydrogen yield [8, 18]. Theoretically, as shown in Eqs. 1 and 2, 4 and 2 mol H_2_ could be produced from 1 mol glucose when the volatile fatty acids are acetic acid and butyric acid, respectively.

1 $${\text{C}}_{ 6} {\text{H}}_{ 1 2} {\text{O}}_{ 6} + {\text{ 2 H}}_{ 2} {\text{O }} \to 2 {\text{ CH}}_{ 3} {\text{COOH}} + {\text{ 2 CO}}_{ 2} + {\text{ 4 H}}_{ 2}$$

2 $${\text{C}}_{ 6} {\text{H}}_{ 1 2} {\text{O}}_{ 6} + {\text{ 2 H}}_{ 2} {\text{O }} \to {\text{ CH}}_{ 3} {\text{CH}}_{ 2} {\text{CH}}_{ 2} {\text{COOH }} + {\text{ 2 CO}}_{ 2} + {\text{ 2 H}}_{ 2}$$

Glucose (usually produced from grains) is the most used feedstock for hydrogen production, which greatly decreases the economic viability of this process. Therefore, the employment of low-value feedstock is becoming a focus. Lignocellulose is the most abundant organic component in the biosphere, with an annual production of 1–5 × 10^13^ kg [19]. However, the incineration of lignocellulose resources such as corn stalks and wheat straw is the main disposal approach, which causes serious environment issues (global warming and air pollution) and wastes resources (10.75–11.25 billion tons per year) [20]. As cellulose and hemicellulose in lignocellulose can be hydrolyzed to glucose and xylose, lignocellulosic biomass (e.g., sugarcane bagasse [SCB], corn cob, corn stalk, and wheat straw) has the potential to be used for hydrogen production [15, 21, 22].

To improve the hydrogen yield from dark fermentation, many studies on process engineering and metabolic engineering of hydrogen-producing organisms have been carried out [16, 22–26]. Thermophilic hydrogen producers are considered ideal hydrogen-producing factories, as higher temperature is beneficial for hydrogen production [27, 28]; for example, thermophilic hydrogen production has higher hydrolysis and reaction rates, lower viscosity and energy consumption, and lower risk of contamination [29].

As shown in our previous studies, *Thermoanaerobacterium aotearoense* SCUT27 shows great potential for conversion of low-value feedstocks to biohydrogen [22]. The *ldh* deletion mutant of SCUT27 could produce 2.28 mol H_2_ from 1 mol xylose in 125-mL serum bottles, and various sugars (mannose, cellobiose, glucose, trehalose, maltose, arabinose, galactose, lactose, fructose, and others.) could be used as carbon sources [22]. The hydrogen production reached 1.86 mol H_2_/mol total sugar when SCB hydrolysate was used as the carbon source, and glucose and xylose in the hydrolysate could be used simultaneously without obvious carbon catabolic repression [6].

**Fig. 1:**
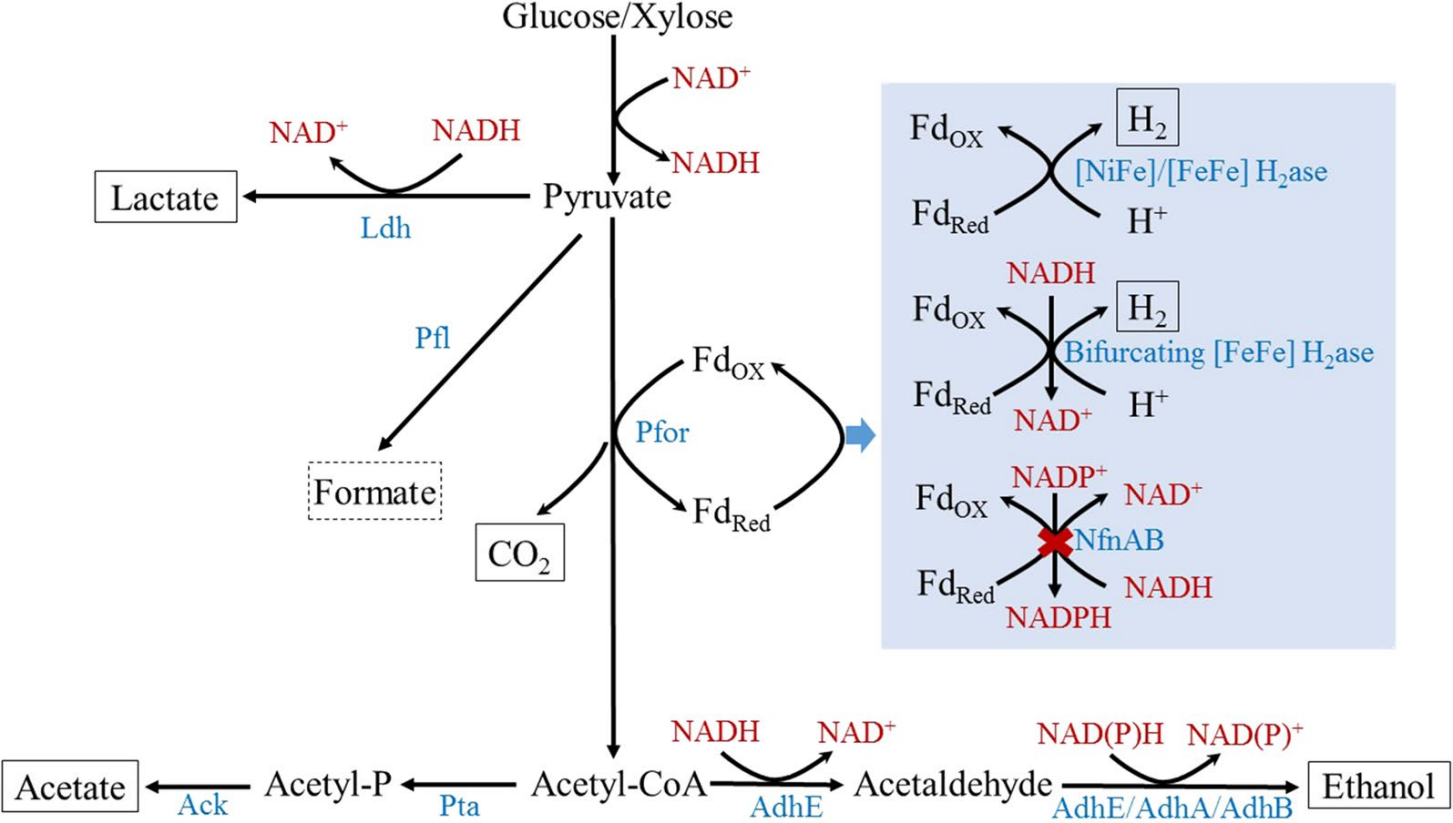
Metabolic pathway in *Thermoanaerobacterium aotearoense* SCUT27. Key enzymes and genes in the pathway: lactate dehydrogenase (*ldh*, accession no. V518_0188); pyruvate formate lyase (*pfl*, accession no. V518_2608); pyruvate:ferredoxin oxidoreductase (*pfor*, accession no. V518_2353); [NiFe]-hydrogenase (*ech*, accession no. V518_1955-1960); [FeFe]-hydrogenase (*hfs*, accession no. V518_1526); bifurcating [FeFe]-hydrogenase, (*hyd*, accession no. V518_0972-0976); NADH-dependent reduced ferredoxin:NADP^+^ oxidoreductase, (*nfnAB*, accession no. V518_0931-0932); phosphate acetyltransferase (*pta*, accession no. V518_1312); acetate kinase, (*ack*, accession no. V518_1311); bifunctional acetaldehyde-CoA/alcohol dehydrogenase, (*adhE*, accession no. V518_0444); alcohol dehydrogenase, (*adhA*, accession no. V518_0933); alcohol dehydrogenase, (*adhB*, accession no. V518_1046). Although PFL has been found in *T. aotearoense* SCUT27, no formate was detected during the fermentation of *T. aotearoense* SCUT27 and SCUT27/*∆nfnAB*

Electron carriers such as ferredoxin and NAD(P)H play an important role in biohydrogen production during dark fermentation. The NADH-dependent reduced ferredoxin:NADP^+^ oxidoreductase (NfnAB) complex containing two subunits was first discovered in *Clostridium kluyveri*, and transfers electrons from reduced ferredoxin and NADH to NADP^+^ (Fd_red_^2−^ + 2 NADP^+^ + NADH + H^+^ → Fd_ox_ + 2 NADPH + NAD^+^) [30]. Hydrogen production in *Thermoanaerobacterium* is NADH-dependent, and blocking pathways competing for NADH or altering the intracellular NADH level could affect hydrogen production [17, 31, 32]. Deletion of *nfnAB* blocks direct NADPH production from NADH, which facilitates the accumulation of NADH-dependent products (i.e., hydrogen, lactic acid and ethanol) (Fig. 1).

Deletion of *nfnAB* had different effects on hydrogen and ethanol formation in different strains of *T. saccharolyticum*, which may be attributed to the cofactor (i.e., NADH or NADPH) specificity of alcohol dehydrogenase. For example, hydrogen production was increased by 46% and 900% after *nfnAB* was deleted from strains JW/SL-YS485 and M1442, respectively, while little effect was observed in strain M0353 [26]. To the best of our knowledge, the cofactor specificity of alcohol dehydrogenase and the role of *nfnAB* in end-product formation by *T. aotearoense* have never been reported so far.

In this work, the effect of *nfnAB* deletion on end-product formation in hydrogen-producing strain *T. aotearoense* SCUT27 was investigated. Then, six types of lignocellulose hydrolysate were selected and evaluated as low-value feedstocks for hydrogen production. Finally, the feasibility of hydrogen production from nonsterilized lignocellulose hydrolysates was investigated.

## Results and discussion

### Hydrogen production using various substrates and intracellular NADH/NAD^+^ ratio of ∆*nfnAB* mutants

**Fig. 2:**
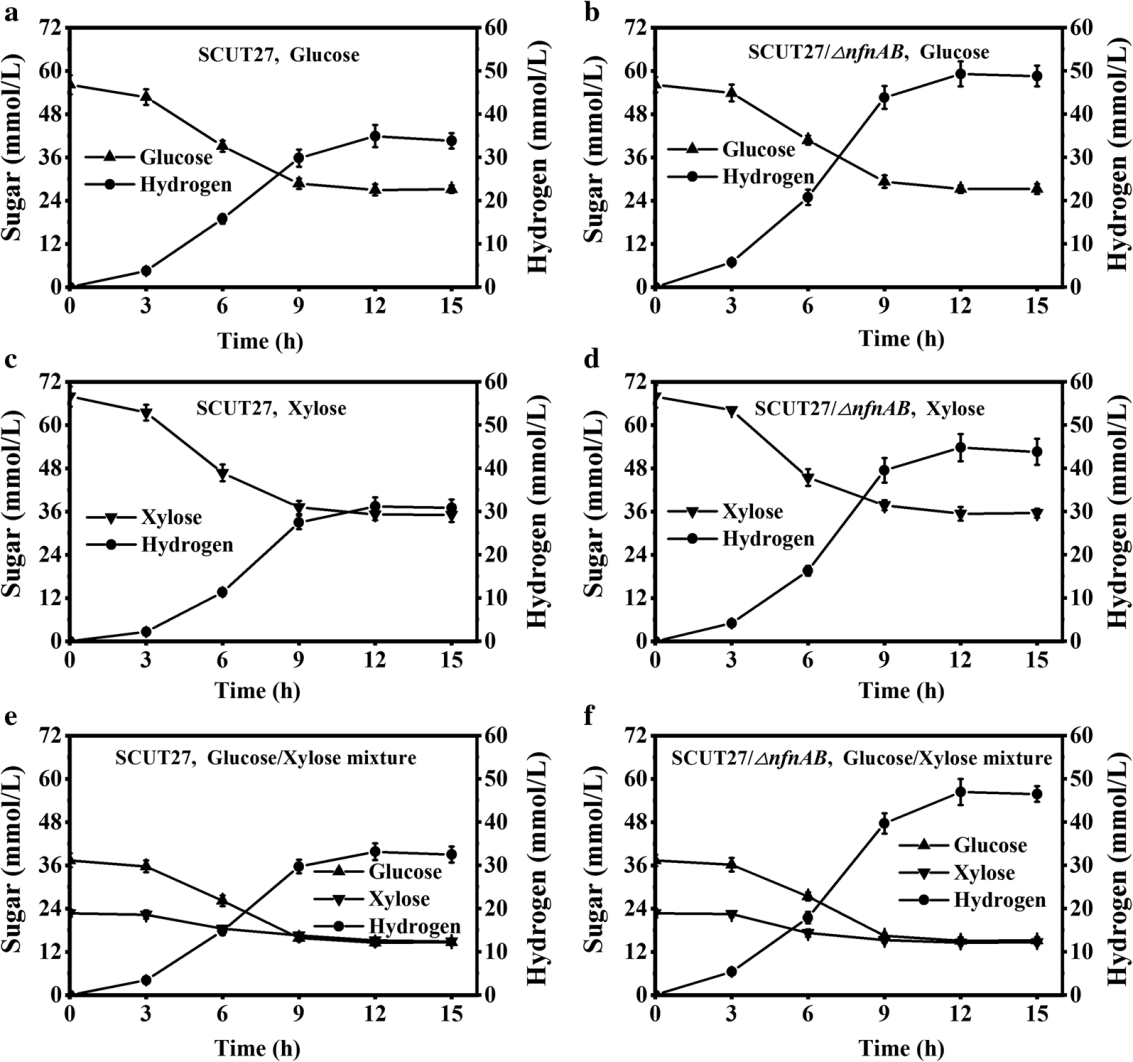
Batch fermentation of SCUT27 and SCUT27/*∆nfnAB* with glucose, xylose or glucose/xylose mixture (2:1) as substrate. Error bars indicate standard error of the mean (*n* = 3). Hydrogen: A,B**; C,D**; E,F**. Substrate consumption and hydrogen production of SCUT27 using glucose (**a**), xylose (**c**) and glucose/xylose mixture (**e**). Substrate consumption and hydrogen production of SCUT/∆*nfnAB* using glucose (**b**), xylose (**d**) and glucose/xylose mixture (**f**)

To evaluate the effect of deletion of *nfnAB* on end-product formation, batch fermentation was first performed in serum bottles with glucose, xylose or glucose/xylose mixture as the substrate. In general, when SCUT27/*∆nfnAB* and its parental strain SCUT27 were inoculated into fresh MTC medium (10 g/L total sugar), no obvious difference was observed in sugar use and acetic acid formation (Fig. 2, Table 1, Additional file 1: Tables S1, S2). The deletion of *nfnAB* resulted in an increase of ethanol production from (8.19 ± 0.66) % to (14.28 ± 1.25)% (*p* < 0.01) and a decrease of lactic acid production from (9.63 ± 0.74)% to (14.62 ± 1.12)% (*p* < 0.05). Hydrogen production by SCUT27/*∆nfnAB* using glucose, xylose and glucose/xylose mixture as substrates increased by (41.1 ± 2.37)% (*p* < 0.01), (43.8 ± 3.18)% (*p* < 0.01) and (38.7 ± 2.65)% (*p* < 0.01), respectively, compared with SCUT27. It should be noted that both SCUT27 and SCUT27/*∆nfnAB* could use glucose and xylose simultaneously during sugar fermentation, although glucose was consumed at a faster rate. Figure 2 shows that the maximum glucose consumption rate of SCUT27 and SCUT27/*∆nfnAB* were (660.71 ± 40.29)% (*p* < 0.01) and (679.31 ± 52.18)% (*p* < 0.01) higher than those of xylose, respectively. To evaluate the effect of pH on metabolite distribution, CaCO_3_ was selected for pH control in serum bottles. As shown in Additional file 1: Table S3, CaCO_3_ had little effect on the product distribution in SCUT27 and SCUT27/*∆nfnAB*, but it increased sugar use because of a buffering effect.

**Table 1:** Comparison of sugar consumption and product formation in batch fermentation of strains SCUT27 and SCUT27/*∆nfnAB* with different carbon sources

Strain	Carbon source	Sugar uptake (mmol/L)	Lactic acid concentration (mmol/L)	Acetic acid concentration (mmol/L)	Ethanol concentration (mmol/L)	Hydrogen concentration (mmol/L)	Hydrogen yield (mol/mol)	Biomass (mmol/L)	Carbon recovery (%)	Electron recovery (%)
SCUT27	Glucose	29.17 ± 2.06	25.44 ± 1.89	15.17 ± 1.33	28.48 ± 2.61	34.92 ± 3.04	1.20 ± 0.09	37.67 ± 2.79	91.59 ± 4.92	96.71 ± 6.98
	Xylose	32.73 ± 2.2	20.78 ± 1.56	13.67 ± 0.67	26.52 ± 1.96	31.14 ± 2.13	0.95 ± 0.06	32.56 ± 2.33	94.67 ± 7.29	93.69 ± 7.16
	G:X = 2:1	30.77 ± 1.95	24.33 ± 1.33	14.67 ± 0.83	27.39 ± 1.52	33.15 ± 1.96	1.12 ± 0.07	33.95 ± 3.26	92.18 ± 6.81	93.59 ± 7.62
SCUT27/*∆nfnAB*	Glucose	28.94 ± 1.78	22 ± 1.67**	14.67 ± 1.01	32.17 ± 2.39**	49.28 ± 2.58**	1.70 ± 0.12**	36.74 ± 2.79	93.72 ± 5.49	98.21 ± 6.75
	Xylose	32.53 ± 1.73	18.78 ± 1.44*	13.5 ± 0.83	28.7 ± 2.39*	44.79 ± 3.78**	1.37 ± 0.08**	32.09 ± 2.33	93.67 ± 6.19	92.38 ± 5.88
	G:X = 2:1	30.14 ± 2.36	20.78 ± 1.56**	14.5 ± 0.67	31.3 ± 1.96*	45.97 ± 2.86**	1.63 ± 0.13**	35.35 ± 2.79	94.75 ± 7.16	96.91 ± 6.25

Asterisks indicate significant differences between SCUT27 and SCUT27/*∆nfnAB* (**p ≤ 0.01; *p ≤ 0.05; *t* test). The carbon in biomass was estimated using the general empirical formula for cell composition of CH_2_N_0.25_O_0.5_ [22]. mol/mol means mol H_2_/mol consumed sugar (glucose and/or xylose)

**Fig. 3:**
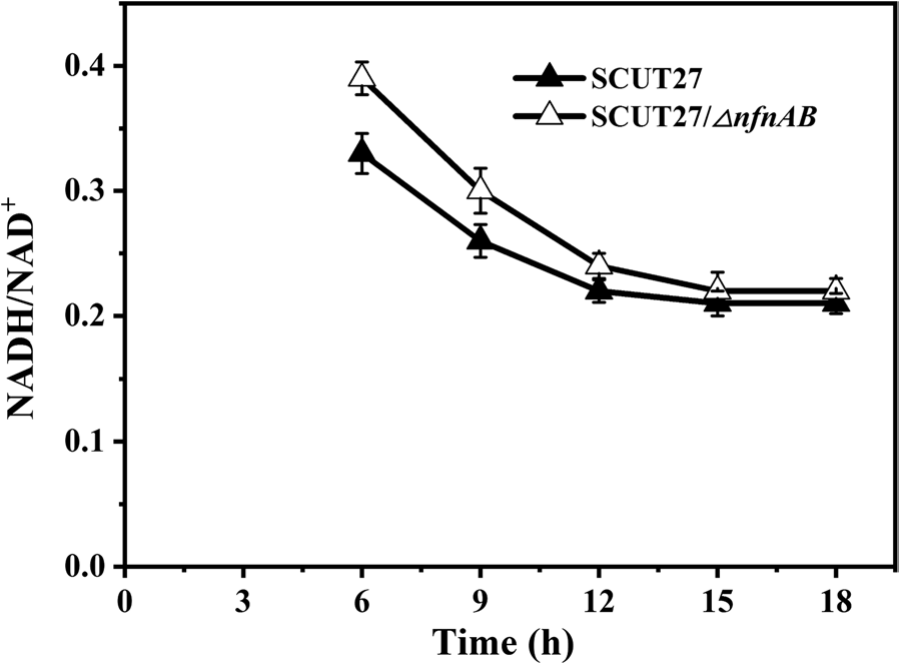
NADH/NAD^+^ ratio in SCUT27 and SCUT27/*∆nfnAB*. Error bars indicate standard error of the mean (*n* = 3). NADH/NAD^+^ ratio: 6 h**, 9 h**, 12 h*

In *T. aotearoense* SCUT27, NADH is consumed by the production of lactic acid, ethanol and hydrogen [22]. Deletion of *nfnAB* from SCUT27 led to a significant increase in hydrogen production. To explain this phenomenon, cells were collected at a 3-h interval from 6 to 18 h after inoculation, then the NADH/NAD^+^ ratio in strains SCUT27 and SCUT27/*∆nfnAB* was determined. As shown in Fig. 3, there was a rapid decrease in the NADH/NAD^+^ ratio from 6 to 12 h, then the NADH/NAD^+^ ratio remained stable beyond 12 h in both strains. However, SCUT27/*∆nfnAB* displayed a much higher NADH/NAD^+^ ratio than the parental strain. For example, at 6 h after inoculation, the NADH/NAD^+^ ratio in SCUT27/*∆nfnAB* was (21.87 ± 2.16)% (*p* < 0.01) higher than that in SCUT27 strain (Fig. 3). The elevated NADH/NAD^+^ ratio disturbed the balance of reducing equivalents, and more NADH was used for hydrogen formation. This result was in accordance with a 46% increase of hydrogen production after *nfnAB* gene deletion in *T. saccharolyticum* strain JW/SL-YS485 [26]. Similarly, downregulation of ferredoxin–NADP^+^ reductase in green alga *Chlamydomonas reinhardtii* increased the electron supply to the hydrogenases, resulting in a 2.5-fold higher hydrogen production rate [33]. In another study, when a fusion gene encoding ferredoxin–hydrogenase was expressed in *C. reinhardtii*, the hydrogen production rate of the mutant was 4.5-fold higher than that of the wild type [34]. Furthermore, our study demonstrated for the first time that *T. aotearoense* SCUT27 primarily uses NADH-dependent aldehyde dehydrogenase/alcohol dehydrogenase for ethanol production, as the decrease of NADPH caused by deletion of *nfnAB* did not significantly affect the formation of ethanol (Table 1). The carbon and electron recoveries of SCUT27 and SCUT27/*∆nfnAB* using glucose, xylose and glucose/xylose mixture as carbon source were between (91.59 ± 4.92)% and (98.21 ± 6.75)%, consistent with the reports of Li and Zhou [22, 35].

### Hydrogen production from lignocellulose hydrolysates in serum bottles

Thermochemical pretreatment of lignocellulose materials has been widely used since it is capable of achieving high solubilization and it is a low-cost process [36]. Both acidic and alkaline solutions are used for the pretreatment of lignocellulose materials. When alkaline-pretreated *Miscanthus* hydrolysate was used as the carbon source, the co-culture of *Caldicellulosiruptor saccharolyticus* and *Thermotoga neapolitana* produced 3.2–3.3 mol H_2_/mol hexose [37]. Many kinds of agricultural residues have been pretreated using dilute acid for biohydrogen production, and dilute acid pretreatment is more successful than other pretreat methods and can improve the hydrolysis of hemicelluloses [38, 39]. Comparison of acid- and alkaline-pretreated grass for hydrogen production indicates that acid pretreatment was better than alkaline pretreatment for hydrogen production [40]. When thermal NH_3_-pretreated rice straw and thermal dilute H_2_SO_4_-pretreated rice straw were used as substrates, the hydrogen production rates of *Thermotoga neapolitana* were 57.64 ± 3.12 and 89.02 ± 5.14 mL H_2_/L/day [38]. Therefore, thermal acid pretreatment was used in this study.

**Fig. 4:**
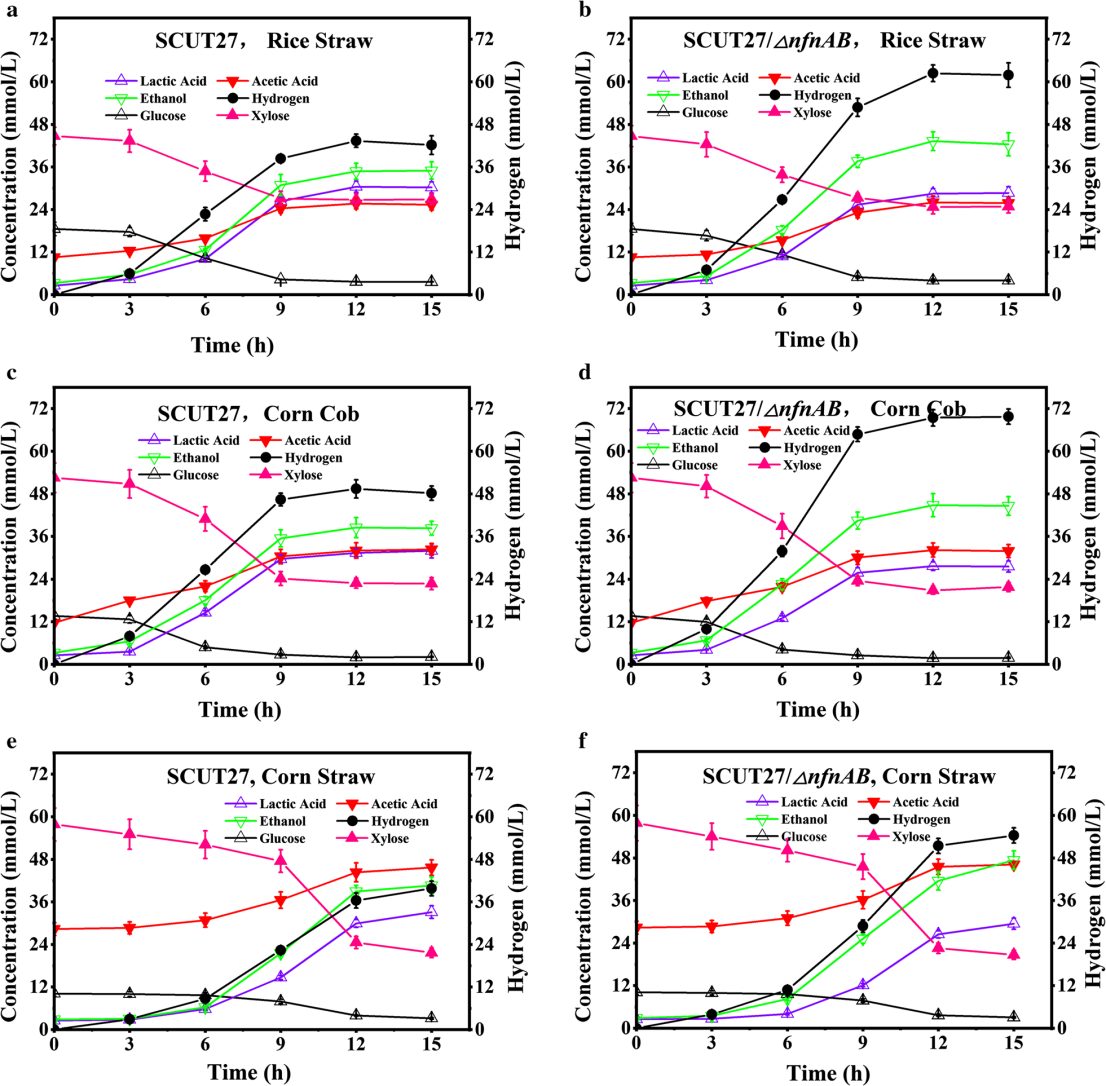
Fermentation kinetics of SCUT27 and SCUT27/*∆nfnAB* in serum bottles using lignocellulose hydrolysate as carbon source. **a** Fermentation of rice straw hydrolysate by SCUT27. **b** Fermentation of rice straw hydrolysate by SCUT27/*∆nfnAB*. **c** Fermentation of corn cob hydrolysate by SCUT27. **d** Fermentation of corn cob hydrolysate by SCUT27/*∆nfnAB*. **e** Fermentation of corn straw hydrolysate by SCUT27. **f** Fermentation of corn straw hydrolysate by SCUT27/*∆nfnAB.* Error bars indicate standard error of the mean (*n* = 3). Hydrogen: A,B**, C,D**, E,F**, G,H**, I,J**, K,L**. Lactic acid: A,B*, C,D**, E,F*, G,H**, I,J**, K,L**. Ethanol: A,B**, C,D**, E,F**, G,H**, I,J**, K,L**

**Fig. 5:**
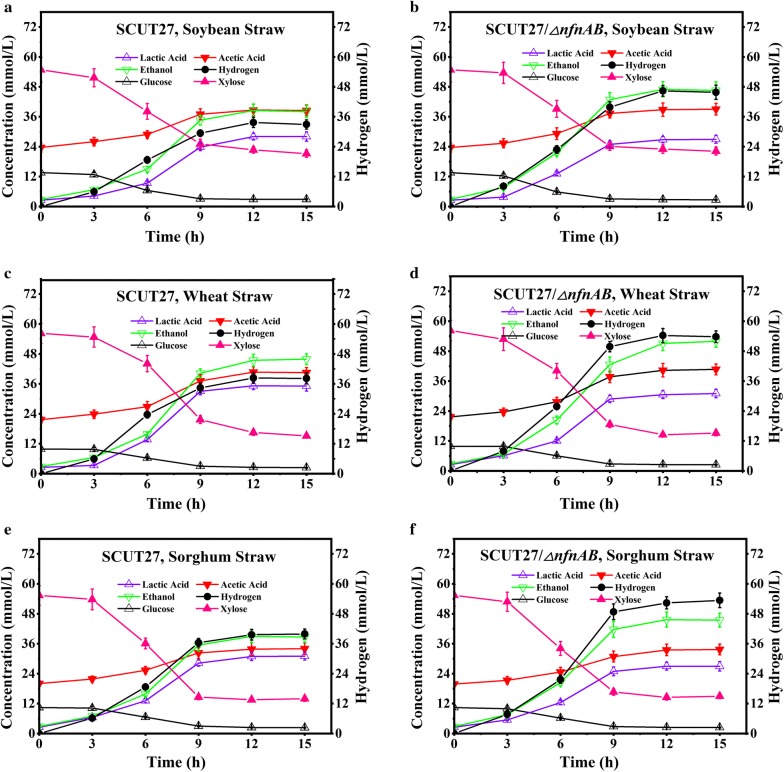
Fermentation kinetics of SCUT27 and SCUT27/*∆nfnAB* in serum bottles using lignocellulose hydrolysate as carbon source. **a** Fermentation of soybean straw hydrolysate by SCUT27. **b** Fermentation of soybean straw hydrolysate by SCUT27/*∆nfnAB*. **c** Fermentation of wheat straw hydrolysate by SCUT27. **d** Fermentation of wheat straw hydrolysate by SCUT27/*∆nfnAB*. **e** Fermentation of sorghum straw hydrolysate by SCUT27. **f** Fermentation of sorghum straw hydrolysate by SCUT27/*∆nfnAB.* Error bars indicate standard error of the mean (*n* = 3). Hydrogen: A,B**, C,D**, E,F**, G,H**, I,J**, K,L**. Lactic acid: A,B*, C,D**, E,F*, G,H**, I,J**, K,L**. Ethanol: A,B**, C,D**, E,F**, G,H**, I,J**, K,L**

Six types of lignocellulose hydrolysate were selected to evaluate their potential application for hydrogen production in serum bottles. The compositions of the hydrolysates after pretreatment are listed in Additional file 1: Tables S4, S5. As expected, compared with SCUT27, strain SCUT27/*∆nfnAB* produced more hydrogen (34.8–43.8)% (*p* < 0.01) with higher productivity and yield (Table 2, Figs. 4 and 5 and Additional file 1: Tables S6–S17). Considering the various lignocellulose hydrolysates, the highest hydrogen yields, 1.21 ± 0.05 mol/mol and 1.65 ± 0.09 mol/mol for SCUT27 and SCUT27/*∆nfnAB*, respectively, were obtained when dilute acid-pretreated corn cob hydrolysate was used as carbon source (Fig. 4c, d), consistent with the results obtained using glucose/xylose mixture (Table 1). In addition, when dilute acid-pretreated rice straw hydrolysate was used as carbon source, the hydrogen yields of SCUT27 and SCUT27/*∆nfnAB* reached 1.16 ± 0.06 mol/mol and 1.48 ± 0.11 mol/mol, respectively (Fig. 4a, b). However, the hydrogen concentration, productivity and yields were much lower when using other hydrolysates (Figs. 4e, f, 5). When dilute acid-pretreated corn straw hydrolysate was used, the fermentation period extended to 15 h, while the fermentation finished in 9–12 h for other hydrolysates (Fig. 4e, f), indicating the relatively high toxicity of corn straw hydrolysate toward *T. aotearoense* SCUT27. As shown in Additional file 1: Table S5, the concentrations of inhibitors in corn straw hydrolysate were much higher than in other hydrolysates, resulting in the longer fermentation period and decreased hydrogen productivity (Table 2). It should be noted that the glucose:xylose ratio in the hydrolysates ranged from 1:1.5 to 1:6.04 (Additional file 1: Table S5). As higher xylose ratio in the hydrolysates may lead to lower hydrogen yield (Table 1), the relatively high hydrogen yield obtained in this study may result from other sugars in the hydrolysates (Additional file 1: Table S4). Based on the results above, corn cob and rice straw hydrolysates were selected for further research.

**Table 2:** Comparison of sugar consumption and product formation by strains SCUT27 and SCUT27/*∆nfnAB* using dilute acid pretreated substrate in serum bottles with 20 mM/L CaCO_3_

Strain	Dilute acid pretreated substrate	Sugar uptake (mmol/L)	Hydrogen concentration (mmol/L)	Hydrogen productivity (mmol/L h)	Hydrogen yield (mol/mol)	Lactic acid production (mmol/L)	Acetic acid production (mmol/L)	Ethanol production (mmol/L)
SCUT27	Rice straw	37.45 ± 1.82	43.38 ± 2.63	3.61 ± 0.22	1.16 ± 0.06	30.44 ± 1.56	15.17 ± 1.17	35 ± 2.39
	Corn cob	41.29 ± 2.25	49.47 ± 2.57	4.12 ± 0.21	1.21 ± 0.05	31.89 ± 2	20.33 ± 2.16	38.48 ± 2.83
	Corn straw	44.56 ± 2.11	39.79 ± 2.11	2.65 ± 0.14	0.91 ± 0.06	33.11 ± 1.78	17.33 ± 1.83	40.65 ± 2.39
	Wheat straw	47.72 ± 2.56	38.35 ± 2.23	3.20 ± 0.19	0.81 ± 0.05	35.11 ± 2	19 ± 2.17	45.43 ± 2.39
	Soybean straw	39.02 ± 1.95	33.69 ± 2.03	2.81 ± 0.17	0.84 ± 0.07	28 ± 1.33	15 ± 1.67	38.48 ± 2.61
	Sorghum straw	40.82 ± 2.37	39.59 ± 1.87	3.63 ± 0.16	0.99 ± 0.04	31 ± 1.56	14 ± 2.5	38.91 ± 2.61
SCUT27/*∆nfnAB*	Rice straw	41.51 ± 2.13*	62.39 ± 3.45**	5.20 ± 0.29**	1.48 ± 0.11**	28.67 ± 1.78*	15.33 ± 1.5	43.26 ± 2.61**
	Corn cob	42.33 ± 2.59	69.76 ± 2.78**	5.81 ± 0.23**	1.65 ± 0.09**	27.67 ± 1.22**	20.33 ± 1.5	44.78 ± 3.26**
	Corn straw	43.98 ± 1.84	54.33 ± 2.15**	3.62 ± 0.14**	1.25 ± 0.07**	29.44 ± 1.56**	17.83 ± 1.67	47.39 ± 2.61**
	Wheat straw	48.35 ± 2.67	54.26 ± 2.83**	4.52 ± 0.24**	1.16 ± 0.05**	31 ± 1.67**	18.67 ± 2.67	51.96 ± 2.39**
	Soybean straw	42.38 ± 2.23*	46.35 ± 2.85**	3.86 ± 0.24**	1.09 ± 0.07**	26.11 ± 2.11*	14.83 ± 1.33	41.52 ± 3.04*
	Sorghum straw	41.69 ± 2.51	53.37 ± 2.97**	4.45 ± 0.25**	1.28 ± 0.09**	26.89 ± 1.89**	13.83 ± 1.67	45.65 ± 3.04**

Asterisks indicate significant differences between SCUT27 and SCUT27/*∆nfnAB* (**p ≤ 0.01; *p ≤ 0.05; *t* test). Biomass was not measured due to the addition of CaCO_3_. mol/mol means mol H_2_/mol consumed sugar (mainly glucose and xylose)

### Hydrogen production from nonsterilized lignocellulose hydrolysates in pH-controlled bioreactor

**Fig. 6:**
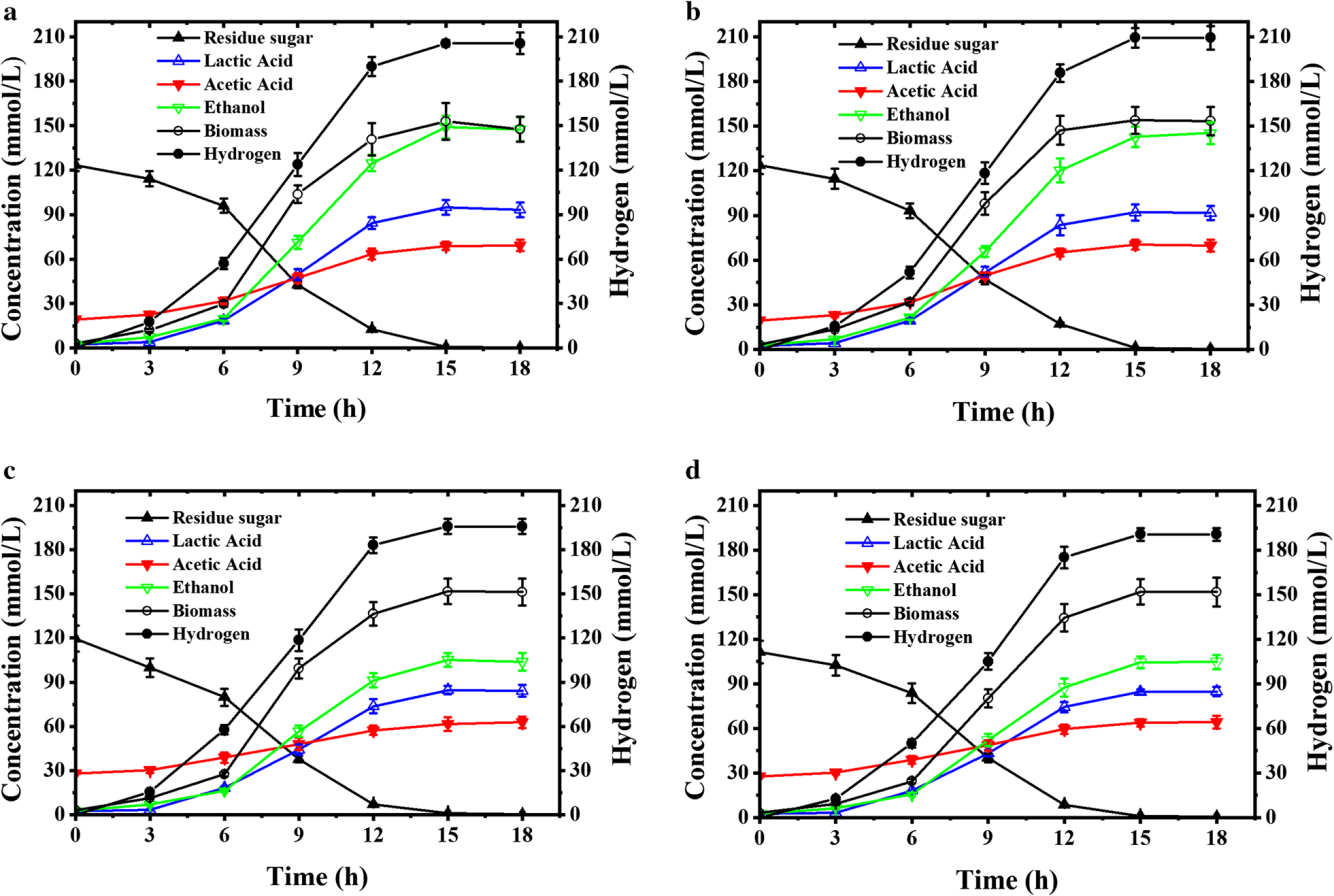
Fermentation kinetics of SCUT27/*∆nfnAB* in pH-controlled bioreactor using lignocellulose hydrolysate as carbon source. **a** Hydrogen fermentation using sterilized rice straw hydrolysate as substrate. **b** Hydrogen fermentation using nonsterilized rice straw hydrolysate as substrate. **c** Hydrogen fermentation using sterilized corn cob hydrolysate as substrate. **d** Hydrogen fermentation using nonsterilized corn cob hydrolysate as substrate

As thermophilic strains have the advantage of lower risk of contamination, hydrogen production by SCUT27/*∆nfnAB* from sterilized (as the control) and nonsterilized hydrolysates of corn cob and rice straw was evaluated in a pH-controlled bioreactor. The fermentation using undiluted hydrolysates was performed at pH 6.0, because the pH value in serum bottles decreased from the initial 6.8 to ~ 5.0 and thus stopped the fermentation due to pH limitation. As shown in Fig. 6 and Additional file 1: Tables S18–S21, all the fermentation processes were finished within 15 h, and no obvious difference in cell density, sugar consumption, or product formation was observed between the fermentation with sterilized and nonsterilized hydrolysates. 16S rDNA sequence analysis showed that there was no bacterial contamination during the fermentation, indicating that it is feasible to produce hydrogen in a nonsterilized fermentation using strains SCUT27 and SCUT27/*∆nfnAB*. In addition, glucose and xylose were totally consumed, which is necessary for efficient hydrogen production from lignocellulosic hydrolysates [41, 42]. For example, the concentration, yield and productivity of hydrogen produced by SCUT27/*∆nfnAB* reached 209.31 mmol/L, 1.77 mol/mol consumed sugar and 13.95 mmol/L h from rice straw hydrolysate, and 195.71 mmol/L, 1.71 mol/mol consumed sugar and 13.04 mmol/L h from corn cob hydrolysate during the fermentation. These values were slightly higher than those using glucose, xylose or mixed sugar as substrate (from 1.37 ± 0.08 to 1.70 ± 0.12 mol/mol consumed sugar) (Table 1), probably due to the presence of other carbon sources other than glucose and xylose in the hydrolysates.

The hydrogen yield and productivity obtained in this study were comparable to or better than those in recent studies of thermophilic hydrogen production from low-value feedstock. For example, Lai et al., reported a hydrogen yield of 1.86 mol/mol consumed sugar using *T. aotearoense* SCUT27/*∆ldh* from SCB hydrolysate, which was slightly higher than the values obtained in this study (1.68–1.79 mol/mol consumed sugar), but the hydrogen productivity (12.43 mmol/L h) of *T. aotearoense* SCUT27/*∆ldh* was lower than that of SCUT27/*∆nfnAB* [43]. In addition, the newly isolated strain *T. thermosaccharolyticum* MJ1 showed a hydrogen yield of 2.52 mol/mol consumed sugar from SCB, but the relatively low hydrogen productivity of 6.55 mmol/L h restricted large-scale production of hydrogen by this strain [21]. The results obtained in the present study indicate that the engineered strain SCUT27/*∆nfnAB* was able to directly use dilute acid-pretreated lignocellulose hydrolysates for hydrogen production with high productivity, yield and substrate use ratio, suggesting that it has great potential to produce hydrogen from nonsterilized lignocellulose hydrolysates.

## Conclusions

The effect of *nfnAB* deletion from *T. aotearoense* SCUT27 on end-product formation was investigated. Deletion of *nfnAB* increased the NADH/NAD^+^ ratio and changed the product distribution, resulting in a significant increase (41.1 ± 2.37)% (*p* < 0.01) in hydrogen production when glucose was the sole carbon source. Our results demonstrate that strain SCUT27/*∆nfnAB* was able to use both glucose and xylose in six types of dilute acid-pretreated biomass hydrolysate for hydrogen production. Furthermore, the possibility of nonsterilized fermentation was verified using corn cob and rice straw hydrolysates. Taken together, these characteristics make *T. aotearoense* SCUT27/*∆nfnAB* a promising target strain to produce hydrogen from lignocellulose hydrolysates.

## Methods

### Strains and cultivation media

The strictly anaerobic bacterium *T. aotearoense* SCUT27 was isolated in Guangdong Province, China [22], and stored in anaerobic tubes at − 80 °C with 25% glycerol as protective agent. Cells were grown in modified MTC medium [22]. The medium contained (per liter): 2.0 g citric acid tripotassium salt, 5.0 g urea, 1.0 g cysteine–HCl monohydrate, 1.25 g citric acid monohydrate, 1.5 g ammonium chloride, 0.1 g ferrous chloride tetrahydrate, 1.0 g sodium sulfate, 1.0 g yeast extract, 1.0 g magnesium chloride hexahydrate, 1.0 g potassium phosphate, 0.2 g calcium chloride, 2.5 g sodium bicarbonate, 0.02 g pyridoxamine dihydrochloride, 0.004 g *p*-aminobenzoic acid, 0.002 g d-biotin, 0.002 g cobalamin, and 0.004 g thiamine chloride. For hydrogen production in a 5-L bioreactor, the 1.0 g/L yeast extract in MTC medium was replaced by 5.0 g/L yeast extract and 2.0 g/L peptone. The effect of yeast extract concentration in the hydrolysate on hydrogen production was negligible (Additional file 1: Table S22). The carbon sources used were glucose, xylose, glucose/xylose mixture or lignocellulosic hydrolysate. Serum bottles were vacuumed and filled with nitrogen three times to guarantee anaerobic conditions, while the bioreactor was sparged with nitrogen for ~ 30 min to reach anaerobiosis. Serum bottle and the bioreactor (containing medium) were sterilized by autoclaving at 121 °C for 30 min. For mutant selection, transformants were grown in modified DSMZ 640 medium [22] with 2.0% agar, in which cellobiose was replaced by xylose. *Escherichia coli* DH5α was used for DNA manipulation and grown in Luria–Bertani medium with 100.0 µg/mL ampicillin.

### Gene cloning and vector construction

The strains, vectors and primers used in this study are listed in Table 3. The genomic DNA of strain SCUT27 was extracted using TIANamp Bacteria DNA Kit (TIANGEN).

**Table 3:** Strains, plasmids and primers used in this study

	Description	Reference or source
Strain		
*T. aotearoense* SCUT27	Wild-type	Our lab
*T. aotearoense* SCUT27/*∆nfnAB*	*nfnAB* deletion mutant of SCUT27	This study
*E. coli DH5α*	Used for plasmid screening and propagation	TianGen
Plasmid		
pBluescript II SK+	Standard cloning vector	Stratagene
pPuKAd	As the template for kanamycin resistance gene amplification	[44]
pBlunAB	Disrupts *nfnAB* with kanamycin resistance gene	This study

Primer	Sequence (5′–3′)	
*nfnA*-F	GGTACCCCTTGCAGGCATTTCTTCC	
*nfnA*-R	GAATTCGAAGGTTGCCCTGTTCACG	
*nfnB*-F	GGATCCCCAACTGTGACTCTGCATC	
*nfnB*-R	GAGCTCAGAAGAAGCAATTGAATCC	
*aph*-F	GGATCCGATAAACCCAGCGAACC	
*aph*-R	GAATTCATCGATACAAATTCCTCGTAGG	
M13-F	CGCCAGGGTTTTCCCAGTCACGAC	
M13-R	AGCGGATAACAATTTCACACAGGA	
probe-F	TTTGCTCGGAAGAGTATGAA	
probe-R	GCCACTTACTTTGCCATCT	

Bacterial plasmid DNA was purified using a TIANprep Mini Plasmid Kit (TIANGEN). The kanamycin resistance gene encoding 3′5′-aminoglycoside phosphotransferase type III (*aph*, Genebank accession no. V01547) was amplified from plasmid pPuKAd stored in our laboratory using primers *aph*-F and *aph*-R, in which *Bam*HI and *Eco*RI sites were added at the 3′-end and the 5′-end, respectively. The purified PCR products were digested with *Bam*HI and *Eco*RI and then ligated into pBluescript II SK (+) (Stratagene, CA, USA) digested with the same enzymes, yielding pBlue-*aph*. To disrupt the genes encoding NfnA (accession no. V518_0931) and NfnB (accession no. V518_0932) in the host chromosome, parts of both genes were replaced by the *aph* gene. Fragments of *nfnA* (810 bp) and *nfnB* (676 bp) were amplified using primers *nfnA-*F/*nfnA-*R and *nfnB*-F/*nfnB*-R, respectively, using SCUT27 genomic DNA as the template. The *nfnA* fragment was digested with *Sac*I and *Bam*HI. The digests were ligated into plasmid pBlue-*aph* digested with the same enzymes to obtain pBlue-*aph*-*nfnA*. The *nfnB* fragment was ligated into pBlue-*aph*-*nfnA*. The final vector pBlunAB was verified by DNA sequence analysis.

### Electro-transformation and transformant screening

pBlunAB was transformed into *T. aotearoense* SCUT27 cells using electroporation, as described previously [45]. The electric-shocked cells were transferred into 1.0 mL of fresh MTC medium and cultivated at 55 °C and 150 rpm for 4 h. The target gene was thus disrupted by homologous recombination [46]. Putative transformants were verified by cultivation on solid DSMZ 640 medium containing kanamycin (50 µg/mL) at 55 °C for 3 days. Genomic DNA was extracted from transformant monoclones and wild-type strain. Transformants were confirmed using PCR with primers *nfn*A-F and *nfn*B-R (Additional file 1: Fig. S1A); a 1.8-kb fragment was amplified from the parental strain SCUT27, while a 3.0-kb fragment was amplified from the *nfnAB*-deletion recombinants. The obtained knockout mutant of *T. aotearoense* SCUT27 was named SCUT27/*∆nfnAB*. Finally, to further verify *nfnAB* deletion, Southern blotting analysis was carried out using primers Pro-F and Pro-R amplified from kanamycin resistance gene (Additional file 1: Fig. S1B); a 2.0-kb fragment was derived from the *nfnAB* deletion mutants, while no bands were observed for the parental strain, owing to the absence of binding sites in the genomic DNAs of SCUT27.

### NADH/NAD^+^ ratio

The NADH/NAD^+^ ratios in wild-type *T. aotearoense* SCUT27 and the *∆nfnAB* mutant were detected using the Amplite™ Colorimetric NAD^+^/NADH Ratio Assay Kit following the manufacturer’s instructions (Amplite, USA). As the OD_600_ value of the sample would reflect the cell density, the biomass was controlled at 2.0 (biomass = 0.46 g/L × OD_600_ × volume). Cells of *T. aotearoense* SCUT27 and SCUT27/*∆nfnAB* were collected at 3-h intervals from 6 to 18 h after inoculation by centrifugation for 15 min at 1.0 × 10^4^×*g* and 0 °C. Then 100 mL of lysis buffer were added to the sample and it was incubated at room temperature for 15 min. After centrifugation at 3000 rpm for 5 min, the supernatant was collected as a test sample. The following steps were performed in clear-bottomed 96-cell microplates. For NAD^+^ extraction assay, 25 μL of NAD^+^ extraction solution was added into the wells containing the test sample. After incubation at room temperature for 15 min, 25 μL of NADH neutralization solution were added to the well. For total NAD^+^ and NADH assay, NAD^+^/NADH control solution was used instead of NAD^+^ extraction solution and NADH neutralization solution. After that, 75 μL of NADH reaction mixture were added into each well containing test sample to make the total volume 150 μL/well. The samples were incubated in the dark room at room temperature for 1 h, followed by measuring the absorbance at 460 nm. Phosphate-buffered saline was used as a blank control. Determinations were carried out in triplicate and all the data were the average of three independent experiments.

### Hydrogen production from different substrates in serum bottles

In this study, dilute sulfuric acid was chosen for pretreatment of the six lignocellulosic biomasses. The acid concentration, temperature and duration of pretreatment were determined depending on our former experiments. The low-value lignocellulose materials (rice straw, corn cob, corn straw, wheat straw, soybean straw, and sorghum straw) used in this study were purchased in Lianyungang City, Jiangsu Province, China. They were milled to 1–2 mm and dried at 65 °C for 24 h before tests. Acid hydrolysis of the feedstock was then performed. The samples were pretreated in dilute sulfuric acid (2.0%, w/v) at 121 °C for 60 min. The hydrolysis was performed in 1-L conical flasks with a solid to liquid ratio of 1:10 (g dry weight to mL). Then the solid residue was removed by centrifugation (10,000×*g*, 15 min). The pH of the supernatant was adjusted to 7.0 with Ca(OH)_2_ and the precipitate formed was removed by centrifugation. Then, vacuum filtration was used to remove solid particles from the supernatant. Soluble sugars (glucose and xylose) and by-products (furfural, 5-methylfurfural, vanillin, etc.) were analyzed using high-performance liquid chromatography (HPLC).

Batch fermentation was performed in MTC medium with 10 g/L glucose, xylose, mixed sugar (glucose:xylose = 2:1), or lignocellulose hydrolysate as the carbon source. Since the optimum sugar concentration for hydrogen production by strain SCUT27 was 10 g/L in serum bottles [22], the hydrolysates were diluted accordingly before inoculation. Then, the fermentation performance of SCUT27 and SCUT27/*∆nfnAB* using these hydrolysates was evaluated. First, SCUT27 and SCUT27/*∆nfnAB* were transferred into 50-mL sterile medium and cultivated at 150 rpm and 55 °C for 12 h. Then, batch fermentation was carried out in serum bottles at 55 °C and 150 rpm, with 5% (v/v) inoculum. For hydrogen production from lignocellulose hydrolysates, 20 mM CaCO_3_ was also added to the serum bottle. Samples were collected at 3-h intervals until the end of the fermentation, and the hydrogen production, residual sugar and other metabolic products in the broth were determined. All treatments were carried out in 125-mL serum bottles with a nitrogen gas headspace. The determinations were carried out in triplicate and the data are the average of three independent experiments.

### Hydrogen production from sterilized and nonsterilized lignocellulose hydrolysates in 5-L bioreactor

Hydrogen fermentation from sterilized and nonsterilized dilute acid-pretreated rice straw and corn cob hydrolysates by SCUT27/*∆nfnAB* were carried out in a 5-L fermentor (BIOSTA A plus, Sartorius Stedim Biotech, Germany) with a working volume of 1 L. The bioreactor was equipped with a stirrer, heating element, and temperature and pH sensors. Seed culture (0.1 L) was transferred into 0.9 L hydrolysate containing the nutrients from modified MTC medium other than the carbon source. The fermentation was performed in anaerobic conditions at 150 rpm and 55 °C. The pH was kept at 6.0 by addition of 5 M of NaOH. The gas phase was collected in a 30-L aluminum foil gasbag (John Long biotech, Beijing, China). Every 3 h, the volume of gas in the gasbag was determined by water displacement. Culture (5 mL) was collected at 3-h intervals, and the residual sugar and products in the supernatant were analyzed using HPLC.

After fermentation, samples were taken from the bioreactor, cells were collected, and the genomic DNAs of the obtained microorganisms were extracted using the TIANamp Bacteria DNA Kit. Then the extracted genomic DNAs were used as template for 16S rDNA amplification. The fragments obtained were verified by DNA sequence analysis.

### Analytical methods

Cell density was determined by measuring turbidity at 600 nm using a spectrophotometer (PERSEE T6, Beijing, China). When hydrolysates were used as carbon source for hydrogen production in serum bottles, the cell density was not monitored due to the interference from CaCO_3_. Soluble sugars and metabolites in the medium were measured using a high-performance liquid chromatography (HPLC) (Waters 2695, Waters, US) equipped with an Amines HPX-87H column (Bio-Rad, Hercules, CA, USA). The column temperature was 60 °C, and 5.0 mM H_2_SO_4_ was used as mobile phase at a flow rate of 0.6 mL/min. The concentrations of by-products produced during the hydrolysis of lignocellulose were analyzed by HPLC equipped with an Agilent HC-C18 column (Agilent, USA). The column temperature was 25 °C, with 10% (v/v) acetonitrile as the mobile phase at a flow of 1.0 mL/min.

Hydrogen concentration was determined by measuring the hydrogen percentage in the headspace or gas stored in the aluminum foil gasbag by gas chromatography (GC; Fuli 9790, Fuli, China) equipped with a flame ionization detector, a thermal conductivity detector, and a TDX-01 column. The column temperature was 60 °C. Nitrogen was used as the carrier gas with a flow rate of 35 mL/min and 10–20 mL sample (including H_2_, CO_2_ and N_2_) was injected into the GC, but only 1.0 mL sample was collected automatically for detection. The percentages of H_2_ and CO_2_ in the sample were calculated according to the corresponding peak areas and standard curves. The standard curves for H_2_ and CO_2_ were obtained as follows: H_2_ or CO_2_ was serially diluted to concentrations of 5%, 10%, 20%, 30%, 40% and 50% with N_2_. The diluted gas samples were injected into the GC and the peak areas of each sample were detected. Then the percentage H_2_, CO_2_ and N_2_ could be obtained from the following equations:

3 $$W_{{{\text{H}}_{ 2} }} = \, - 1. 3 7 { } + \, A_{{{\text{H}}_{ 2} }} \times { 6}. 5 3\times 10^{ - 6} ,$$

4 $$W_{{{\text{CO}}_{ 2} }} = { 7}.0 2 { } + \, A_{{{\text{CO}}_{ 2} }} \times { 9}. 7 1\times 10^{ - 5} ,$$

5 $$W_{{{\text{N}}_{ 2} }} = { 1}00 \, - \, W_{{{\text{H}}_{ 2} }} - \, W_{{{\text{CO}}_{ 2} }} ,$$

where *A* and *W* are the peak area and the percentage of the corresponding gas.

The initial *N*_2_ quantity ($n_{{{\text{N}}_{ 2} }}$) in the serum bottle headspace gas was evaluated as 4.31 mmol ($n_{{{\text{N}}_{ 2} }}$ = *pv*/*RT*, *p* = 0.14 Pa, *v* = 75 mL, *T* = 298.15 K, *R* = 8.314 m^3^ Pa/K mol).

H_2_ quantity ($n_{{{\text{H}}_{ 2} }}$, mmol) in the serum bottles was deduced as:

6 $$n_{{{\text{H}}_{ 2} }} = n_{{{\text{N}}_{ 2} }} \times \, R_{{{\text{H}}_{ 2} /{\text{N}}_{ 2} }} = n_{{{\text{N}}_{ 2} }} \times W_{{{\text{H}}_{ 2} }} / \, W_{{{\text{N}}_{ 2} }} ,$$

where $R_{{{\text{H}}_{ 2} /{\text{N}}_{ 2} }}$ is the molar ratio of H_2_ to N_2_ in the detected sample.

H_2_ quantity ($n_{{{\text{H}}_{ 2} }}$, mmol) in the bioreactor was calculated as:

7 $$n_{{{\text{H}}_{ 2} }} = pV_{{{\text{H}}_{ 2} }} /RT = pW_{{{\text{H}}_{ 2} }} \times V_{\text{g}} /RT,$$

where *V*_g_ is the volume of the gas collected in the gasbag (*p* = 0.1 MPa, *T* = 298.15 K, *R* = 8.314 m^3^ Pa/K mol).

H_2_ yield ($Y_{{{\text{H}}_{ 2} }}$, mol H_2_/mol sugar) was calculated as:

8 $$Y_{{{\text{H}}_{ 2} }} = n_{{{\text{H}}_{ 2} }} / n_{\text{sugar}} ,$$

where *n*_sugar_ is the amount of sugar consumed during the fermentation, in mmol.

For hydrogen production using hydrolysates or glucose/xylose mixture, the quantity of each consumed sugar was calculated separately and summed to give total consumed sugar.

Carbon recovery calculation was based on Li [22]:

9 $$\begin{aligned} C_{\text{t}} & = 0. 4 {\text{ sugar }} + 0.4 {\text{ lactate }} + 0.6 {\text{ acetate }} \\ & \quad + 0. 78 {\text{ ethanol }} + 0.47 {\text{ CDW,}} \end{aligned}$$

where *C*_t_ = total carbon; sugar = glucose, xylose or glucose/xylose mixture; CDW = cell dry weight. All units are g/L.

Electron recovery calculation was based on Zhou [35]. The general empirical formula for cell composition was CH_2_N_0.25_O_0.5_. Carbon contained in yeast extract was not included in the carbon and electron recovery calculations.

### Statistical analysis

Statistical tests were performed using Statistical Package for the Social Sciences (SPSS ver. 19; IBM Corporation, USA). The data presented in the manuscript and Additional file are the means and standard deviations (**p ≤ 0.01; *0.01 ≤ *p* ≤ 0.05; *t* test).

## Supplementary information

**Additional file 1: Fig. S1.** Verification of *nfnAB* deletion mutants. (A) PCR amplification analysis of *nfnAB* deletion mutants and their parental strains. M: DL5,000 DNA ladder; lane 1 and lane 2: SCUT27/*∆nfnAB*; lane 3 and lane 4: SCUT27. (B) Southern blots of *nfnAB* deletion mutant and the parental strains. M: DNA molecular weight marker; lane 1: vector pBlunAB; lane 2 and lane 3: SCUT27; lane 4 and lane 5: SCUT27/*∆nfnAB*. **Table S1.** Hydrogen production and sugar use of SCUT27 with glucose, xylose and glucose/xylose mixture. **Table S2.** Hydrogen production and sugar use of SCUT27/*∆nfnAB* with glucose, xylose and glucose/xylose mixture. **Table S3.** The effect of CaCO_3_ addition on products distribution (mol/mol glucose) for SCUT27 and SCUT27/*∆nfnAB*. **Table S4.** Concentration of arabinose, mannose and galactose in different hydrolysates. **Table S5.** Composition of various dilute acid-pretreated lignocellulose hydrolysates. **Table S6.** Raw data for fermentation of SCUT27 using rice straw hydrolysate in serum bottles. **Table S7.** Raw data for fermentation of SCUT27/*∆nfnAB* using rice straw hydrolysate in serum bottles. **Table S8.** Raw data for fermentation of SCUT27 using corn cob hydrolysate in serum bottles. **Table S9.** Raw data for fermentation of SCUT27/*∆nfnAB* using corn cob hydrolysate in serum bottles. **Table S10.** Raw data for fermentation of SCUT27 using corn straw hydrolysate in serum bottles. **Table S11.** Raw data for fermentation of SCUT27/*∆nfnAB* using corn straw hydrolysate in serum bottles. **Table S12.** Raw data for fermentation of SCUT27 using soybean straw hydrolysate in serum bottles. **Table S13.** Raw data for fermentation of SCUT27/*∆nfnAB* using soybean straw hydrolysate in serum bottles. **Table S14.** Raw data for fermentation of SCUT27 using wheat straw hydrolysate in serum bottles. **Table S15.** Raw data for fermentation of SCUT27/*∆nfnAB* using wheat straw hydrolysate in serum bottles. **Table S16.** Raw data for fermentation of SCUT27 using sorghum straw hydrolysate in serum bottles. **Table S17.** Raw data for fermentation of SCUT27/*∆nfnAB* using sorghum straw hydrolysate in serum bottles. **Table S18.** Raw data for fermentation of SCUT27/*∆nfnAB* using sterilized rice straw hydrolysate in 5-L bioreactor. **Table S19.** Raw data for fermentation of SCUT27/*∆nfnAB* using nonsterilized rice straw hydrolysate in 5-L bioreactor. **Table S20.** Raw data for fermentation of SCUT27/*∆nfnAB* using sterilized corn cob hydrolysate in 5-L bioreactor. **Table S21.** Raw data for fermentation of SCUT27/*∆nfnAB* using nonsterilized corn cob hydrolysate in 5-L bioreactor. **Table S22.** Effect of yeast extract concentration on sugar consumption and product distribution (mol/mol substrate) of *T. aotearoense* SCUT27.

## Data Availability

Data will be made available from the corresponding author on reasonable request.

## Abbreviations

NfnAB: NADH-dependent reduced ferredoxin:NAD^+^ oxidoreductase
SCB: sugarcane bagasse hydrolysate
HPLC: high-performance liquid chromatography
