# Supplementary material for: Engineered Thermoanaerobacterium aotearoense with nfnAB knockout for improved hydrogen production from lignocellulose hydrolysates
Source: Biotechnol Biofuels. 2019 Sep 10;12:214. doi: 10.1186/s13068-019-1559-8 (PMC6737674; doi:10.1186/s13068-019-1559-8)
Supplement: Supplementary file 1 — Additional file 1: Fig. S1. Verification of nfnAB deletion mutants. (A) PCR amplification analysis of nfnAB deletion mutants and their parental strains. M: DL5,000 DNA ladder; lane 1 and lane 2: SCUT27/∆nfnAB; lane 3 and lane 4: SCUT27. (B) Southern blots of nfnAB deletion mutant and the parental strains. M: DNA molecular weight marker; lane 1: vector pBlunAB; lane 2 and lane 3: SCUT27; lane 4 and lane 5: SCUT27/∆nfnAB. Table S1. Hydrogen production and sugar use of SCUT27 with glucose, xylose and glucose/xylose mixture. Table S2. Hydrogen production and sugar use of SCUT27/∆nfnAB with glucose, xylose and glucose/xylose mixture. Table S3. The effect of CaCO3 addition on products distribution (mol/mol glucose) for SCUT27 and SCUT27/∆nfnAB. Table S4. Concentration of arabinose, mannose and galactose in different hydrolysates. Table S5. Composition of various dilute acid-pretreated lignocellulose hydrolysates. Table S6. Raw data for fermentation of SCUT27 using rice straw hydrolysate in serum bottles. Table S7. Raw data for fermentation of SCUT27/∆nfnAB using rice straw hydrolysate in serum bottles. Table S8. Raw data for fermentation of SCUT27 using corn cob hydrolysate in serum bottles. Table S9. Raw data for fermentation of SCUT27/∆nfnAB using corn cob hydrolysate in serum bottles. Table S10. Raw data for fermentation of SCUT27 using corn straw hydrolysate in serum bottles. Table S11. Raw data for fermentation of SCUT27/∆nfnAB using corn straw hydrolysate in serum bottles. Table S12. Raw data for fermentation of SCUT27 using soybean straw hydrolysate in serum bottles. Table S13. Raw data for fermentation of SCUT27/∆nfnAB using soybean straw hydrolysate in serum bottles. Table S14. Raw data for fermentation of SCUT27 using wheat straw hydrolysate in serum bottles. Table S15. Raw data for fermentation of SCUT27/∆nfnAB using wheat straw hydrolysate in serum bottles. Table S16. Raw data for fermentation of SCUT27 using sorghum straw hydrolysate in serum bottles. Table S [file 13068_2019_1559_MOESM1_ESM.docx]

**Fig. S1.** Verification of *nfnAB* deletion mutants. (A) PCR amplification analysis of *nfnAB* deletion mutants and their parental strains. M: DL5,000 DNA ladder; lane 1 and lane 2: SCUT27/*△nfnAB*; lane 3 and lane 4: SCUT27. (B) Southern blots of *nfnAB* deletion mutant and the parental strains. M: DNA molecular weight marker; lane 1: vector pBlunAB; lane 2 and lane 3: SCUT27; lane 4 and lane 5: SCUT27/*△nfnAB*. **Table S1.** Hydrogen production and sugar use of SCUT27 with glucose, xylose and glucose/xylose mixture. **Table S2.** Hydrogen production and sugar use of SCUT27/*△nfnAB* with glucose, xylose and glucose/xylose mixture. **Table S3.** The effect of CaCO_3_ addition on products distribution (mol/mol glucose) for SCUT27 and SCUT27/*△nfnAB*. **Table S4.** Concentration of arabinose, mannose and galactose in different hydrolysates. **Table S5.** Composition of various dilute acid-pretreated lignocellulose hydrolysates. **Table S6.** Raw data for fermentation of SCUT27 using rice straw hydrolysate in serum bottles. **Table S7.** Raw data for fermentation of SCUT27/*△nfnAB* using rice straw hydrolysate in serum bottles. **Table S8.** Raw data for fermentation of SCUT27 using corn cob hydrolysate in serum bottles. **Table S9.** Raw data for fermentation of SCUT27/*△nfnAB* using corn cob hydrolysate in serum bottles. **Table S10.** Raw data for fermentation of SCUT27 using corn straw hydrolysate in serum bottles. **Table S11.** Raw data for fermentation of SCUT27/*△nfnAB* using corn straw hydrolysate in serum bottles. **Table S12.** Raw data for fermentation of SCUT27 using soybean straw hydrolysate in serum bottles. **Table S13.** Raw data for fermentation of SCUT27/*△nfnAB* using soybean straw hydrolysate in serum bottles. **Table S14.** Raw data for fermentation of SCUT27 using wheat straw hydrolysate in serum bottles. **Table S15.** Raw data for fermentation of SCUT27/*△nfnAB* using wheat straw hydrolysate in serum bottles. **Table S16.** Raw data for fermentation of SCUT27 using sorghum straw hydrolysate in serum bottles. **Table S17.** Raw data for fermentation of SCUT27/*△nfnAB* using sorghum straw hydrolysate in serum bottles. **Table S18.** Raw data for fermentation of SCUT27/*△nfnAB* using sterilized rice straw hydrolysate in 5-L bioreactor. **Table S19.** Raw data for fermentation of SCUT27/*△nfnAB* using nonsterilized rice straw hydrolysate in 5-L bioreactor. **Table S20.** Raw data for fermentation of SCUT27/*△nfnAB* using sterilized corn cob hydrolysate in 5-L bioreactor. **Table S21.** Raw data for fermentation of SCUT27/*△nfnAB* using nonsterilized corn cob hydrolysate in 5-L bioreactor. Table S22. Effect of yeast extract concentration on sugar consumption and product distribution (mol/mol substrate) of *T. aotearoense* SCUT27.

**
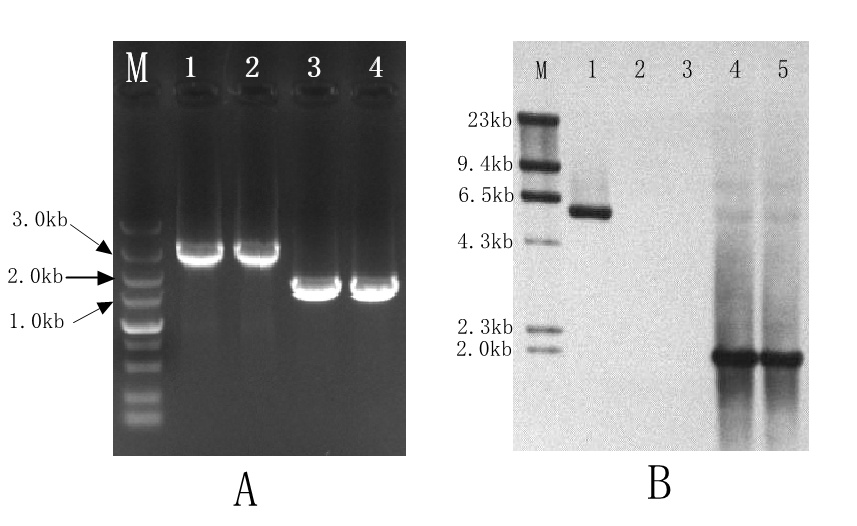
**

**Fig. S1.** Verification of *nfnAB* deletion mutants. (A) PCR amplification analysis of *nfnAB* deletion mutants and their parental strains. M: DL5,000 DNA ladder; lane 1 and lane 2: SCUT27/*△nfnAB*; lane 3 and lane 4: SCUT27. (B) Southern blots of *nfnAB* deletion mutant and the parental strains. M: DNA molecular weight marker; lane 1: vector pBlunAB; lane 2 and lane 3: SCUT27; lane 4 and lane 5: SCUT27/*△nfnAB*.

**Table S1.** Hydrogen production and sugar use of SCUT27 with glucose, xylose and glucose/xylose mixture.

| Time  (h) | Glucose  (mmol/L) | H_2_ (Glucose)  (mmol/L) | Xylose  (mmol/L) | H_2_ (Xylose)  (mmol/L) | Glucose  (mmol/L) | Xylose  (mmol/L) | H_2_ (Mixture)  (mmol/L) |
| --- | --- | --- | --- | --- | --- | --- | --- |
| 0 | 56.17±2.61 | 0 | 67.93±2.8 | 0 | 37.39±1.94 | 22.73±1.07 | 0 |
| 3 | 52.72±2.17 | 3.75±0.21 | 63.47±2.2 | 2.17±0.11 | 35.72±1.61 | 22.33±1.13 | 3.41±0.27 |
| 6 | 39.11±1.5 | 15.77±1.05 | 46.73±2.33 | 11.33±0.84 | 26.17±1.5 | 18.4±0.8 | 14.88±1.03 |
| 9 | 28.72±1.44 | 29.84±1.97 | 37.13±1.8 | 27.49±1.55 | 15.89±0.94 | 16.53±0.46 | 29.72±1.58 |
| 12 | 27±1.56 | 34.92±2.54 | 35.2±1.6 | 31.14±2.13 | 14.56±0.89 | 15.13±0.53 | 33.15±1.96 |
| 15 | 27.17±1.06 | 33.82±1.77 | 35.13±2.07 | 30.82±2 | 14.67±0.61 | 14.86±0.86 | 32.49±1.84 |

**Table S2.** Hydrogen production and sugar use of SCUT27/*△nfnAB* with glucose, xylose and glucose/xylose mixture.

| Time  (h) | Glucose  (mmol/L) | H_2_ (Glucose)  (mmol/L) | Xylose  (mmol/L) | H_2_ (Xylose)  (mmol/L) | Glucose  (mmol/L) | Xylose  (mmol/L) | H_2_ (Mixture)  (mmol/L) |
| --- | --- | --- | --- | --- | --- | --- | --- |
| 0 | 56.17±2.11 | 0 | 67.93±3.13 | 0 | 37.38±1.61 | 22.73±1.27 | 0 |
| 3 | 53.83±2.33 | 5.77±0.33 | 64.13±0.46 | 4.21±0.31 | 36.17±1.89 | 22.47±0.8 | 5.53±0.21 |
| 6 | 40.78±1.27 | 20.86±1.77 | 45.4±2.33 | 16.29±1.12 | 27.39±1.17 | 17.2±1.06 | 17.29±1.32 |
| 9 | 29.28±1.72 | 43.26±2.64 | 37.73±1.4 | 39.85±2.85 | 16.44±0.72 | 15.27±0.73 | 39.46±2.36 |
| 12 | 27.22±1.06 | 49.28±2.89 | 35.4±1.87 | 44.79±3.32 | 15.6±1 | 14.6±0.4 | 46.97±2.89 |
| 15 | 27.28±1.39 | 48.22±2.37 | 35.53±1.26 | 44.14±3.04 | 15.17±0.83 | 14.67±0.73 | 46.49±2.25 |

**Table S3.** The effect of CaCO_3_ addition on products distribution (mol/mol glucose) for SCUT27 and SCUT27/*△nfnAB*.

| CaCO_3_  concentration  (mM) | SCUT27 | | | | | SCUT27/*△nfnAB* | | | | |
| --- | --- | --- | --- | --- | --- | --- | --- | --- | --- | --- |
|  | Glucose utilized (mmol) | Lactic acid  (mol/mol) | Acetic acid  (mol/mol) | Ethanol  (mol/mol) | Hydrogen  (mol/mol) | Glucose utilized (mmol) | Lactic acid  (mol/mol) | Acetic acid  (mol/mol) | Ethanol  (mol/mol) | Hydrogen  (mol/mol) |
| 0 | 29.5±2.33 | 0.82±0.07 | 0.47±0.04 | 0.93±0.07 | 1.26±0.08 | 29.17±1.6 | 0.75±0.05 | 0.46±0.04 | 1.07±0.09 | 1.71±0.12 |
| 10 | 38.59±2.7 | 0.83±0.06 | 0.46±0.03 | 0.95±0.08 | 1.22±0.07 | 37.91±1.78 | 0.74±0.04 | 0.47±0.02 | 1.09±0.07 | 1.69±0.14 |
| 20 | 40±3.01 | 0.84±0.07 | 0.48±0.04 | 0.95±0.07 | 1.23±0.09 | 39.69±1.35 | 0.74±0.04 | 0.47±0.03 | 1.10±0.08 | 1.75±0.15 |
| 30 | 40.12±2.27 | 0.83±0.07 | 0.46±0.03 | 0.94±0.06 | 1.27±0.1 | 39.63±1.9 | 0.74±0.05 | 0.48±0.02 | 1.08±0.08 | 1.73±0.11 |
| 40 | 40.18±1.6 | 0.84±0.05 | 0.46±0.04 | 0.95±0.08 | 1.26±0.12 | 39.51±2.09 | 0.73±0.06 | 0.47±0.03 | 1.06±0.07 | 1.76±0.14 |

**Table S4.** Concentration of arabinose, mannose and galactose in different hydrolysates.

| Substrate | Arabinose  (g/L) | Mannose  (g/L) | Galactose  (g/L) |
| --- | --- | --- | --- |
| Corn straw | 0.09±0.01 | ND | 0.05±0.01 |
| [Soybean straw](http://dict.youdao.com/w/soybean%20straw/#keyfrom=E2Ctranslation) | 0.1±0.02 | 0.02±0.01 | 0.02±0.01 |
| Wheat straw | 0.12±0.01 | ND | 0.03±0.01 |
| Sorghum straw | 0.09±0.01 | 0.02±0.01 | 0.03±0.01 |
| Corn cob | 0.12±0.02 | ND | 0.02±0.01 |
| Rice straw | 0.11±0.02 | 0.03±0.01 | ND |

ND: Not Detected.

**Table S5.** Composition of various dilute acid-pretreated lignocellulose hydrolysates.

| Substrate | Glucose (g/L) | Xylose (g/L) | Acetic acid (g/L) | Furfural (g/L) | 5-HMF (g/L) | Ferulic acid (mg/L) | Vanillin  (mg/L) | Syringaldehyde (mg/L) | Coumaric acid (mg/L) |
| --- | --- | --- | --- | --- | --- | --- | --- | --- | --- |
| Corn straw | 2.89±0.14 | 17.47±0.94 | 3.24±0.22 | 0.21±0.01 | 0.33±0.06 | 87.53±9.14 | 57.11±4.48 | 27.56±1.25 | 58.74±3.77 |
| Soybean straw | 3.72±0.22 | 14.07±0.83 | 2.38±0.16 | 0.13±0.01 | 0.15±0.02 | 55.13±4.21 | 41.33±3.9 | 19.28±2.3 | 33.25±1.96 |
| Wheat straw | 2.77±0.13 | 12.83±0.73 | 1.97±0.07 | 0.11±0.01 | 0.09±0.01 | 65.17±6.32 | 28.76±3.15 | 22.68±3.17 | 41.74±3.17 |
| Sorghum straw | 3.53±0.1 | 16.24±1.05 | 2.24±0.11 | 0.16±0.02 | 0.18±0.02 | 77.36±4.69 | 37.68±4.22 | 35.22±3.11 | 25.24±2.63 |
| Corn cob | 5.46±0.13 | 16.64±1.13 | 2.06±0.17 | 0.15±0.02 | 0.22±0.01 | 46.73±5.15 | 39.66±4.8 | 21.49±2.58 | 28.76±1.97 |
| Rice straw | 10.12±1.04 | 15.21±0.95 | 1.41±0.13 | 0.19±0.01 | 0.15±0.02 | 53.66±4.18 | 68.72±5.71 | 19.66±1.37 | 36.99±2.55 |

**Table S6.** Raw data for fermentation of SCUT27 using rice straw hydrolysate in serum bottles.

| Time  (h) | Residue sugar  (mmol/L) | Lactic acid  (mmol/L) | Hydrogen (mmol/L) | Acetic acid  (mmol/L) | Ethanol  (mmol/L) |
| --- | --- | --- | --- | --- | --- |
| 0 | 62.83±2.5 | 2.56±0.11 | 0 | 10.5±0.5 | 3.26±0.22 |
| 3 | 61±2.39 | 4.33±0.11 | 5.9±0.56 | 12.33±0.5 | 5.65±0.22 |
| 6 | 45.06±1.44 | 10.1±0.55 | 22.68±1.88 | 15.83±0.83 | 12.61±0.24 |
| 9 | 31.39±1.5 | 26.33±1.22 | 38.37±0.98 | 24.33±1.33 | 30.87±2.97 |
| 12 | 30.39±0.83 | 30.44±1.55 | 43.38±1.87 | 25.67±1.5 | 34.78±2.26 |
| 15 | 30.44±0.89 | 30.22±1.55 | 42.18±2.63 | 25.33±1.5 | 35±2.43 |

**Table S7.** Raw data for fermentation of SCUT27/*△nfnAB* using rice straw hydrolysate in serum bottles.

| Time  (h) | Residue sugar  (mmol/L) | Lactic acid  (mmol/L) | Hydrogen (mmol/L) | Acetic acid  (mmol/L) | Ethanol  (mmol/L) |
| --- | --- | --- | --- | --- | --- |
| 0 | 63.83±2.72 | 2.56±0.11 | 0 | 10.5±0.5 | 3.26±0.22 |
| 3 | 59.56±2.44 | 4.11±0.11 | 6.928±0.07 | 11.33±0.5 | 5.22±0.22 |
| 6 | 42.83±1.94 | 10.78±0.44 | 26.79±1 | 15.33±1 | 18.26±1.09 |
| 9 | 29.94±1.44 | 25.33±0.89 | 52.77±2.54 | 23.17±1.5 | 37.61±1.74 |
| 12 | 27.67±0.94 | 28.44±1.44 | 62.39±2.33 | 26±1.83 | 43.26±2.61 |
| 15 | 27.72±1.78 | 28.67±1.78 | 61.88±3.45 | 25.83±1.5 | 42.39±3.26 |

**Table S8.** Raw data for fermentation of SCUT27 using corn cob hydrolysate in serum bottles.

| Time  (h) | Residue sugar  (mmol/L) | Lactic acid  (mmol/L) | Hydrogen (mmol/L) | Acetic acid  (mmol/L) | Ethanol  (mmol/L) |
| --- | --- | --- | --- | --- | --- |
| 0 | 66.73±3 | 2.56±0.11 | 0 | 11.67±0.5 | 3.26±0.22 |
| 3 | 63.47±2.13 | 3.56±0.11 | 7.9±0.36 | 18±0.83 | 6.52±0.65 |
| 6 | 45.67±2.53 | 14.56±0.55 | 26.68±1.18 | 22±1.5 | 18.04±1.08 |
| 9 | 26.93±0.87 | 29.67±1 | 46.37±1.78 | 30.33±2 | 35.43±2.39 |
| 12 | 24.53±0.47 | 31.33±1.22 | 49.38±2.57 | 32±2.17 | 38.47±2.82 |
| 15 | 24.27±0.73 | 31.89±2 | 48.18±2.03 | 32.33±1.67 | 38.26±1.95 |

**Table S9.** Raw data for fermentation of SCUT27/*△nfnAB* using corn cob hydrolysate in serum bottles.

| Time  (h) | Residue sugar  (mmol/L) | Lactic acid  (mmol/L) | Hydrogen (mmol/L) | Acetic acid  (mmol/L) | Ethanol  (mmol/L) |
| --- | --- | --- | --- | --- | --- |
| 0 | 66.73±3.53 | 2.56±0.11 | 0 | 11.67±0.67 | 3.26±0.22 |
| 3 | 63.4±3.06 | 4.11±0.11 | 9.928±0.45 | 17.83±1 | 6.74±0.43 |
| 6 | 42±2.64 | 13±0.67 | 31.79±1.51 | 21.83±1.17 | 22.61±1.52 |
| 9 | 25.87±1.4 | 25.78±1.44 | 64.77±2.04 | 30±1.83 | 40.43±2.39 |
| 12 | 23.13±1 | 27.67±1.22 | 69.39±2.33 | 32.14±2 | 44.78±3.26 |
| 15 | 23.27±0.6 | 27.56±1.56 | 69.76±2.15 | 31.83±1.84 | 44.56±2.61 |

**Table S10.** Raw data for fermentation of SCUT27 using corn straw hydrolysate in serum bottles.

| Time  (h) | Residue sugar  (mmol/L) | Lactic acid  (mmol/L) | Hydrogen (mmol/L) | Acetic acid  (mmol/L) | Ethanol  (mmol/L) |
| --- | --- | --- | --- | --- | --- |
| 0 | 68.87±3.4 | 2.56±0.11 | 0 | 28.33±1.83 | 2.83±0.22 |
| 3 | 67.93±2.8 | 2.78±0.11 | 2.9±0.06 | 28.67±1.67 | 3.04±0.22 |
| 6 | 65.67±2.2 | 5.78±0.33 | 8.68±0.78 | 30.83±2 | 6.3±0.22 |
| 9 | 56.93±2.07 | 14.67±0.55 | 22.37±1.15 | 36.5±2.33 | 21.52±0.87 |
| 12 | 27.2±1.8 | 29.89±1.11 | 36.38±2.11 | 44.33±2.67 | 38.91±1.74 |
| 15 | 23.2±1 | 33.11±1.78 | 39.79±2.06 | 45.67±2.17 | 40.65±2.39 |

**Table S11.** Raw data for fermentation of SCUT27/*△nfnAB* using corn straw hydrolysate in serum bottles.

| Time  (h) | Residue sugar  (mmol/L) | Lactic acid  (mmol/L) | Hydrogen (mmol/L) | Acetic acid  (mmol/L) | Ethanol  (mmol/L) |
| --- | --- | --- | --- | --- | --- |
| 0 | 68.87±3.07 | 2.56±0.11 | 0 | 28.33±1.83 | 2.83±0.22 |
| 3 | 66.8±3.47 | 2.67±0.11 | 3.928±0.05 | 28.67±1.67 | 3.48±0.22 |
| 6 | 64.73±2.33 | 4±0.22 | 10.79±0.81 | 31.01±2 | 8.26±0.43 |
| 9 | 53.13±1.4 | 12.11±0.44 | 28.77±1.74 | 36.17±2.5 | 25.22±1.3 |
| 12 | 26.6±1.27 | 26.44±1 | 51.39±2.13 | 45.5±2.17 | 41.52±2.61 |
| 15 | 23.67±0.93 | 29.44±1.56 | 54.33±2.15 | 46.17±0.67 | 47.39±2.61 |

**Table S12.** Raw data for fermentation of SCUT27 using soybean straw hydrolysate in serum bottles.

| Time  (h) | Residue sugar  (mmol/L) | Lactic acid  (mmol/L) | Hydrogen (mmol/L) | Acetic acid  (mmol/L) | Ethanol  (mmol/L) |
| --- | --- | --- | --- | --- | --- |
| 0 | 67.6±3.47 | 2.56±0.11 | 0 | 23.67±1.83 | 2.82±0.22 |
| 3 | 62.8±3.07 | 4.22±0.11 | 5.9±0.26 | 26±1.67 | 6.73±0.22 |
| 6 | 44.46±1.73 | 9.33±0.67 | 18.68±1.35 | 28.83±1.5 | 15±1.08 |
| 9 | 28.26±0.26 | 23.78±1.44 | 29.37±1.28 | 37±2.17 | 34.57±1.95 |
| 12 | 26.53±1.2 | 28±1.33 | 33.69±2.03 | 38.67±2.33 | 38.48±2.61 |
| 15 | 26.6±0.93 | 28±1.89 | 32.88±1.85 | 38.5±2.17 | 38.04±2.39 |

**Table S13.** Raw data for fermentation of SCUT27/*△nfnAB* using soybean straw hydrolysate in serum bottles.

| Time  (h) | Residue sugar  (mmol/L) | Lactic acid  (mmol/L) | Hydrogen (mmol/L) | Acetic acid  (mmol/L) | Ethanol  (mmol/L) |
| --- | --- | --- | --- | --- | --- |
| 0 | 67.6±2.8 | 2.56±0.11 | 0 | 23.67±1.83 | 2.83±0.22 |
| 3 | 63.26±3.73 | 3.78±0.11 | 8.6±0.39 | 25.33±1.83 | 7.61±0.34 |
| 6 | 45.87±2.86 | 13.11±0.67 | 22.97±2.17 | 29.17±2.33 | 21.52±1.09 |
| 9 | 27.73±1.4 | 24.89±1.44 | 41.84±2.82 | 37.33±1.83 | 42.83±2.83 |
| 12 | 19.6±0.87 | 26.78±1.22 | 46.35±2.85 | 38.83±2.67 | 46.96±3.04 |
| 15 | 19.47±1.27 | 26.89±1.56 | 45.39±2.85 | 39±2.33 | 46.52±3.48 |

**Table S14.** Raw data for fermentation of SCUT27 using wheat straw hydrolysate in serum bottles.

| Time  (h) | Residue sugar  (mmol/L) | Lactic acid  (mmol/L) | Hydrogen (mmol/L) | Acetic acid  (mmol/L) | Ethanol  (mmol/L) |
| --- | --- | --- | --- | --- | --- |
| 0 | 65.26±2.93 | 2.56±0.11 | 0 | 21.67±1.33 | 2.83±0.22 |
| 3 | 61.47±2.06 | 3.33±0.11 | 5.9±0.31 | 23.83±1.67 | 6.52±0.22 |
| 6 | 46.33±2.13 | 13.67±0.67 | 23.68±1.36 | 26.83±2 | 15.87±0.65 |
| 9 | 21.6±1.53 | 33±1.44 | 34.37±1.88 | 37.17±2.5 | 40.22±1.74 |
| 12 | 16.53±0.73 | 35.22±1.56 | 38.38±2.17 | 40.67±2.17 | 45.43±2.39 |
| 15 | 16.27±0.87 | 35.11±2 | 38.18±2.23 | 40.5±2 | 45.87±2.17 |

**Table S15.** Raw data for fermentation of SCUT27/*△nfnAB* using wheat straw hydrolysate in serum bottles.

| Time  (h) | Residue sugar  (mmol/L) | Lactic acid  (mmol/L) | Hydrogen  (mmol/L) | Acetic acid  (mmol/L) | Ethanol  (mmol/L) |
| --- | --- | --- | --- | --- | --- |
| 0 | 65.27±2.8 | 2.56±0.11 | 0 | 21.67±1.33 | 2.83±0.22 |
| 3 | 61.27±2.2 | 6.11±0.22 | 7.928±0.45 | 23.67±1.5 | 6.74±0.22 |
| 6 | 44.53±1.93 | 12±0.56 | 25.79±1.21 | 27.66±1.83 | 20.43±1.52 |
| 9 | 21.07±1.4 | 28.78±1.44 | 49.77±2.04 | 37.67±2.33 | 42.61±3.04 |
| 12 | 15.93±0.8 | 30.56±1.56 | 54.26±2.83 | 40.33±2.67 | 51.08±2.82 |
| 15 | 15.8±1.27 | 31±1.67 | 53.76±2.35 | 40.67±2.16 | 51.95±2.39 |

**Table S16.** Raw data for fermentation of SCUT27 using sorghum straw hydrolysate in serum bottles.

| Time  (h) | Residue sugar  (mmol/L) | Lactic acid  (mmol/L) | Hydrogen (mmol/L) | Acetic acid  (mmol/L) | Ethanol  (mmol/L) |
| --- | --- | --- | --- | --- | --- |
| 0 | 66.93±2.8 | 2.56±0.11 | 0 | 20±1.17 | 2.83±0.22 |
| 3 | 63.27±2.33 | 6.44±0.22 | 6.2±0.38 | 21.83±1.17 | 6.96±0.43 |
| 6 | 45.67±2.06 | 13.11±0.78 | 18.68±1.13 | 25.33±1.5 | 15.87±0.87 |
| 9 | 27.13±1.4 | 28.22±1.22 | 36.37±1.69 | 32.33±2.33 | 35.43±2.39 |
| 12 | 24.93±1.27 | 30.89±1.67 | 39.59±2.15 | 33.83±2.5 | 38.91±2.61 |
| 15 | 25.06±1.07 | 31±1.56 | 39.88±1.97 | 34±2.33 | 38.69±2.17 |

**Table S17.** Raw data for fermentation of SCUT27/*△nfnAB* using sorghum straw hydrolysate in serum bottles.

| Time  (h) | Residue sugar  (mmol/L) | Lactic acid  (mmol/L) | Hydrogen (mmol/L) | Acetic acid  (mmol/L) | Ethanol  (mmol/L) |
| --- | --- | --- | --- | --- | --- |
| 0 | 66.93±3.4 | 2.56±0.11 | 0 | 19.83±0.83 | 2.82±0.22 |
| 3 | 63.6±2.8 | 5.44±0.33 | 7.66±0.51 | 21.33±1.5 | 7.6±0.43 |
| 6 | 46.4±2.2 | 12.44±0.78 | 21.67±1.65 | 24.66±1.83 | 20.43±1.3 |
| 9 | 25.73±1.27 | 24.89±1.78 | 48.77±3.15 | 30.83±2.16 | 41.73±3.26 |
| 12 | 24.2±1.13 | 27±1.56 | 52.35±2.44 | 33.5±2.33 | 45.65±3.04 |
| 15 | 24.33±1 | 26.89±1.89 | 53.37±2.95 | 33.67±2.17 | 45.43±2.82 |

**Table S18.** Raw data for fermentation of SCUT27/*△nfnAB* using sterilized rice straw hydrolysate in 5-L bioreactor.

| Time | Residue sugar | Lactic Acid | Hydrogen | Acetate | Ethanol |
| --- | --- | --- | --- | --- | --- |
| (h) | (mmol/L) | (mmol/L) | (mmol/L) | (mmol/L) | (mmol/L) |
| 0 | 123.82±6.89 | 2.44±0.11 | 0 | 19.33±0.5 | 2.61±0.22 |
| 3 | 114.16±5.05 | 4±0.33 | 17.64±1.28 | 22.67±1 | 7.39±0.22 |
| 6 | 96.04±4.83 | 18.56±1.67 | 57.12±3.67 | 31.83±2.33 | 19.78±1.52 |
| 9 | 42.76±2.28 | 49.56±3.56 | 123.79±7.98 | 47.33±3.16 | 71.3±4.56 |
| 12 | 12.77±0.78 | 84.44±4.16 | 189.84±6.55 | 63.5±3.5 | 124.78±5.43 |
| 15 | 0.35±0.02 | 93.26±4.97 | 205.52±2.36 | 68.83±2.83 | 149.13±7.61 |

**Table S19.** Raw data for fermentation of SCUT27/*△nfnAB* using nonsterilized rice straw hydrolysate in 5-L bioreactor.

| Time | Residue sugar | Lactic Acid | Hydrogen | Acetate | Ethanol |
| --- | --- | --- | --- | --- | --- |
| (h) | (mmol/L) | (mmol/L) | (mmol/L) | (mmol/L) | (mmol/L) |
| 0 | 117.68±5.94 | 2.44±0.11 | 0 | 19.5±0.33 | 2.61±0.22 |
| 3 | 108.66±6.71 | 4.33±0.22 | 15.7±1.22 | 23.17±1.5 | 6.96±0.43 |
| 6 | 93.13±4.97 | 19.43±1.33 | 51.84±3.95 | 31.67±1.83 | 21.52±1.52 |
| 9 | 46.99±3.1 | 21.78±3.78 | 118.44±7.33 | 49.67±2.17 | 65.87±3.48 |
| 12 | 17.11±1.14 | 83.56±6.78 | 185.58±5.81 | 65.17±2.67 | 120.14±8.04 |
| 15 | 1.17±0.04 | 92.11±5.44 | 209.31±6.57 | 70.5±3.17 | 129.78±6.96 |

**Table S20.** Raw data for fermentation of SCUT27/*△nfnAB* using sterilized corn cob hydrolysate in 5-L bioreactor.

| Time | Residue sugar | Lactic Acid | Hydrogen | Acetate | Ethanol |
| --- | --- | --- | --- | --- | --- |
| (h) | (mmol/L) | (mmol/L) | (mmol/L) | (mmol/L) | (mmol/L) |
| 0 | 116.41±7.65 | 2.44±0.11 | 0 | 28.14±0.5 | 2.61±0.22 |
| 3 | 99.94±6.38 | 3.33±0.22 | 15.4±1.53 | 30.33±1.83 | 6.96±0.43 |
| 6 | 79.74±5.92 | 18.33±0.88 | 57.68±3.11 | 38.83±3.5 | 16.52±0.87 |
| 9 | 37.79±2.16 | 44.22±3.56 | 118.52±7.25 | 48±4.16 | 56.74±3.91 |
| 12 | 7.23±0.55 | 73.67±4.78 | 183.02±5.37 | 63.5±3.17 | 105.22±4.57 |
| 15 | 1.17±0.09 | 84.67±2.89 | 195.71±5.17 | 61.5±4.67 | 103.22±6.09 |

**Table S21**. Raw data for fermentation of SCUT27/*△nfnAB* using nonsterilized corn cob hydrolysate in 5-L bioreactor.

| Time | Residue sugar | Lactic Acid | Hydrogen | Acetate | Ethanol |
| --- | --- | --- | --- | --- | --- |
| (h) | (mmol/L) | (mmol/L) | (mmol/L) | (mmol/L) | (mmol/L) |
| 0 | 111.41±7.65 | 2.44±0.11 | 0 | 27.67±0.83 | 2.61±0.22 |
| 3 | 102.39±6.94 | 3.11±0.22 | 12.78±1.14 | 30.17±1.17 | 6.3±0.39 |
| 6 | 83.7±6.63 | 18.15±0.43 | 49.92±2.87 | 38.14±3.22 | 15.65±1.08 |
| 9 | 40.24±2.91 | 43.52±2.11 | 105.08±5.69 | 48.67±3.5 | 52.17±4.13 |
| 12 | 8.53±0.66 | 74.31±3.56 | 175.1±7.29 | 59.5±3.16 | 104.56±3.91 |
| 15 | 1.04±0.03 | 84.56±1.89 | 190.63±4.26 | 63.83±4.33 | 104.78±4.78 |

**Table S22**. Effect of the yeast extract concentration on sugar consumption and product distribution (mol/mol substrate)

of *T. aotearoense* SCUT27.

| Yeast extract  concentration  (g/L) | Sugar  uptake  (g/L) | Hydrogen  production  (mol/mol) | Lactic acid production  (mol/mol) | Acetic acid production  (mol/mol) | Ethanol  production  (mol/mol) | Biomass  production  (mol/mol) |
| --- | --- | --- | --- | --- | --- | --- |
| 0 | 1.35±0.08 | 1.14±0.01 | 0.77±0.06 | 0.33±0.02 | 0.92±0.08 | 1.59±0.15 |
| 1 | 5.22±0.37** | 1.14±0.01 | 0.83±0.05* | 0.48±0.04** | 0.94±0.07 | 1.02±0.08** |
| 5 | 5.19±0.42** | 1.15±0.02 | 0.82±0.06* | 0.48±0.03** | 0.94±0.07 | 1.05±0.07** |

Note: batch fermentation with mixed sugar (6.66 g/L glucose + 3.33 g/L xylose).
